# Hypertension in rural communities in Delta State, Nigeria: Prevalence, risk factors and barriers to health care

**DOI:** 10.4102/phcfm.v7i1.875

**Published:** 2015-12-17

**Authors:** Mary I. Ofili, Busisiwe P. Ncama, Benn Sartorius

**Affiliations:** 1 Department of Nursing Science, Delta State University, Nigeria; 2 School of Nursing and Public Health, University of KwaZulu-Natal, South Africa

## Abstract

**Introduction:**

Hypertension is a global health challenge and its prevalence is increasing rapidly amongst adults in many African countries. Some studies on the prevalence and risk factors of hypertension have been conducted in Nigeria, but none within Delta State. We assessed the prevalence of hypertension and associated risk factors amongst adults in three villages in the Ibusa community in Delta State, Nigeria.

**Method:**

Homesteads were randomly selected and all consenting adults (≥ 18 years of age) were recruited for this cross-sectional study (134 individuals: 48 men, 86 women). Sociodemographic data and anthropometric measurements (weight, height and abdominal circumference) were recorded. Diagnosis of hypertension was based on blood pressure ≥ 140/90 mmHg.

**Result:**

Hypertension prevalence in this rural community was 44%. Results from one village (Ogboli: 82%) and ethnic group (Ibo: 50%) were significantly higher than in others in the same variable category. Multivariate logistic regression analysis suggested increasing age, increasing body mass index and high salt intake as prominent risk factors for hypertension. Lack of funds and equipment shortage in clinics were most often reported as barriers to health care.

**Conclusion:**

A nutritional education programme to promote low-cholesterol and low-salt diets is recommended to specifically target people in higher-risk areas and of higher-risk ethnicity. Local barriers to accessing health care need to be addressed.

## Abstrait

**Hypertension dans les Communautés rurales du Delta State, Nigeria: Prévalence, Facteurs de Risques et Obstacles aux Soins de Santé.**

**Introduction:**

L’hypertension est un problème de santé global dont la prévalence augmente rapidement chez les adultes de nombreux pays africains. Quelques études ont été faites sur la prévalence et les facteurs de risques de l’hypertension au Nigeria, mais aucune dans le Delta State. Nous avons examiné la prévalence de l’hypertension et les facteurs de risques connexes parmi les adultes de trois villages dans la communauté Ibusa du Delta State, au Nigeria.

**Méthode:**

Nous avons sélectionné des maisons au hasard et tous les adultes consentants (≥ 18 ans) ont été recrutés pour cette étude transversale (134 individus: 48 hommes, 86 femmes). Nous avons enregistré les données sociodémographiques et les mesures anthropométriques (poids, taille et circonférence abdominale). Le diagnostic d’hypertension a été fait en mesurant la tension artérielle ≥ 140/90 mmHg.

**Résultat:**

Dans cette communauté rurale la prévalence d’hypertension était de 44%. Les résultats d’un village (Ogboli: 82%) et du groupe ethnique (Ibo: 50%) étaient bien plus élevés que dans les autres de la même catégorie variable. L’analyse de la régression logistique multivariée a montré que les facteurs majeurs de risque d’hypertension sont l’âge, l’augmentation de l’indice de masse corporelle et une consommation élevée de sel. Le manque de fonds et de matériel dans les dispensaires étaient le plus souvent cités comme obstacles aux soins de santé.

**Conclusion:**

Un programme d’éducation nutritionnelle encourageant des régimes faibles en cholestérol et pauvre en sel a été recommandé pour les gens des régions et ethnicités à haut risques. Il faudra se concentrer sur les obstacles locaux d’accès aux soins de santé.

## Introduction

Hypertension, also known as high blood pressure, is one of the most common non-communicable diseases affecting a large percentage of adult individuals worldwide. The World Health Organization (WHO) estimates that more than 30 million people in Africa present with hypertension.^1^ The WHO also predicts that if the condition is not curbed by 2020, three-quarters of all deaths in Africa could be attributable to hypertension.^1^ More recently, studies^2,3^ have revealed that hypertension (in many African countries and rural settings) is on the increase, with estimated prevalence rates ranging between 20% and 40%.

In Nigeria, depending on the study population, type of measurement and cut-off value used for defining hypertension, the prevalence of hypertension in a rural setting ranges from 13.5% to 46.4%, compared with 8.1% – 42.0% in urban settings.^4^ In three rural communities of Ife North, a local government area (LGA) of Osun State in south-western Nigeria, the prevalence of hypertension was found to be 26.4%, suggesting an increasing prevalence of the disease.^5^ In Abia State, hypertension was found to be high in both rural and urban settings.^6^ In the Niger Delta region of Nigeria, the prevalence of hypertension in this rural community was found to be 20.2%.^7^ Studies have also reported increasing age and body mass index (BMI) (i.e. obesity) as the most strongly related risk factors associated with hypertension.^5,6,8^

Despite the various efforts and initiatives in developing countries to prevent or manage hypertension, there are still some barriers limiting optimal outcomes. These barriers can exist at the level of patients, staff or health system and administration.^9^ Examples of such barriers include a lack of funding, which affects basic day-to-day operation of health care facilities, scarcity of and difficult access to health care centres in a community, staff shortages in health care centres, shortage of drugs in clinics and dispensaries, limited availability of equipment and insufficient maintenance, and insufficient patient health education and communication in clinics.^9,10,11,12^

However, most of the studies regarding the prevalence and risk factors of hypertension in Nigeria focused on urban communities or rural areas in south-western and south-eastern Nigeria. To our knowledge, no studies have yet assessed prevalence of hypertension and associated predictors or risk factors in the South-South region of Nigeria (Delta State).

## Research methods and design

### Study area and population

**FIGURE 1:**
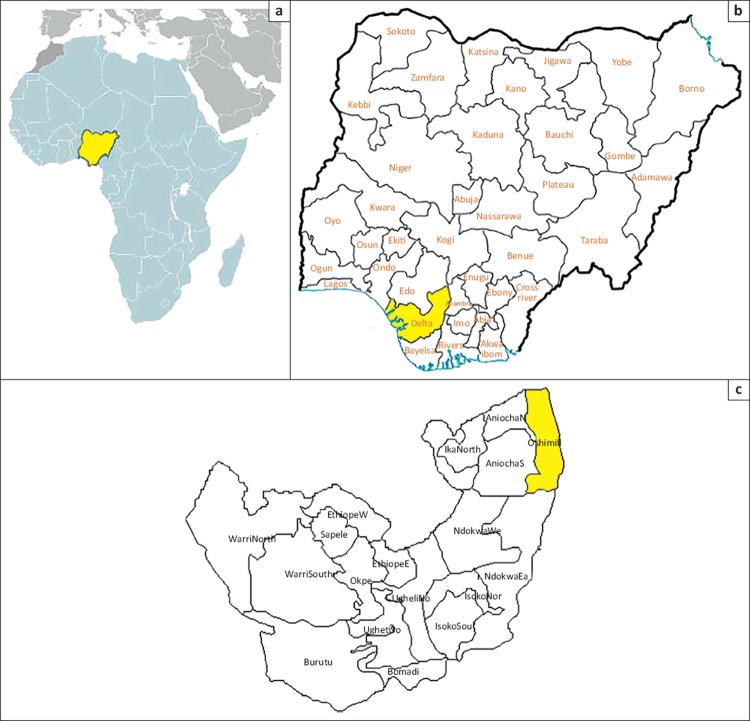
Maps of the study area and community, showing (a) location of the country (Nigeria) in West Africa, (b) the relative location of Delta State (yellow) in the southern part of Nigeria and (c) the location of the Ibusa community (yellow) in Oshimili North.

Delta State is one of six states in the oil-rich South-South region of Nigeria and consists of 25 LGAs; 10 of these are urban and 15 are rural. The study was conducted in the Ibusa community in Oshimili North LGA, one of the 15 rural LGAs (Figure 1). Ten villages form part of the Ibusa community, with an overall population of 20 166.^13^ All these villages have a primary school, but only four villages have a secondary school. There are three primary health care centres (only two are functional), one general hospital and one maternal and child health centre in this community. There is no formal water supply infrastructure, although some villages have functional boreholes. The villages are linked by untarred roads and the residents are predominantly farmers and hunters. The study population comprised adult residents (18 years or older) of the Idinisagba, Umuodafe and Ogboli villages.

### Study design, sampling method and sample size

An epidemiological survey (homestead to homestead) was conducted. The three villages (Idinisagba, Umuodafe and Ogboli) were randomly selected as study sites. Simple random sampling were used to select homesteads for sampling, resulting in eight homesteads being sampled in Idinisagba, ten in Umuodafe and six in Ogboli. The population across the 24 homesteads was 210, of which a final sample of 134 were eligible for inclusion and willing to participate. Of the total sample, 45 participants were from Idinisagba, 67 from Umuodafe and 22 from Ogboli.

### Study procedures

The questionnaire used in the survey collected various sociodemographic data on the respondents’ general health care practices and their family history of hypertension. It also contained questions related to potential risk factors associated with raised blood pressure and obstacles to health care for the management of hypertension.

Blood pressure and anthropometric measurements (weight, height and abdominal circumference) were also recorded. Blood pressure was measured using a digital automatic blood pressure monitor (OMRON MX2, Omron Health Care), which has been clinically validated to provide blood pressure readings directly comparable with those of standard mercury sphygmomanometers. A portable electronic scale (Hana, Omron Health Care) was used to measure body weight and a tape measure (Sandex Powerlock-P5NE) for height and abdominal circumference measurement. All instruments were reset to zero after individual measurements.

Body weight was recorded to the nearest 0.1 kg (after removal of shoes, heavier clothing and pocket contents). Height (without shoes and head scarf) and abdominal circumference were recorded to the nearest 0.5 cm. Abdominal circumference was measured midway between the last rib and the iliac crest and measurements ≥ 102 cm and ≥ 88 cm were regarded as indicative of abdominal obesity in male and female participants, respectively. The BMI categories used in this study were based on the WHO classification:^14,15,16^

- 18.5 kg/m^2^ – 24.9 kg/m^2^ regarded as normal weight
- 25.0 kg/m^2^ – 29.9 kg/m^2^ regarded as overweight
- ≥ 30 kg/m^2^ regarded as obese.

Blood pressure was measured in the left arm, with the subject in a seated position and after at least five minutes’ rest. Cuffs of an appropriate size for arm circumference were used. Three blood pressure readings were taken per subject, with at least three minutes between measurements. The average of the three readings was used in the analysis. Hypertension (stage 1) is commonly defined as a systolic pressure ≥ 140 mm Hg or diastolic pressure ≥ 90 mmHg as measured in a clinical setting,^17^ together with systolic and diastolic averages exceeding 135 mmHg and 85 mmHg, respectively, during ambulatory or home blood pressure monitoring. In our study, hypertension was defined either as blood pressure measuring ≥ 140/90 mmHg or as self-reported use of antihypertensive medication.^18^

### Data analysis

Data were processed and analysed using the statistical software package Stata (verison 13, StataCorp.). Significant associations between various explanatory variables and hypertension were assessed using the standard Pearson’s chi-square (χ^2^) test. If an expected cell count in the crosstabulation had less than five observations (sparse numbers), exact methods (namely Fisher’s exact test) were used instead. Bivariate and multivariate-adjusted logistic regressions were also used to assess the influence of various explanatory variables or confounders on hypertension status. Factors with a P-value of < 0.2 based on the bivariate associations were selected for entry into the multivariate-adjusted logistic model. An explanatory variable with an adjusted P-value of < 0.05 was deemed statistically significant. A goodness-of-fit test was performed to measure adequacy of the final model.

### Ethical considerations

Ethical approval for this study was granted by the Humanities and Social Sciences Research Ethics Committee of the University of KwaZulu-Natal, Durban (protocol reference number: HSS/0525/013D). Gatekeeper’s permission was also obtained from the ruler of Ibusa community (the Obuzor of Ibusa).

## Results

Table 1 presents a summary of the characteristics of the study participants (n = 134). Half the sample (67 participants) were from Umuodafe village. The age range of respondents was between 20 and 91 years, with a mean age and standard deviation of 52.6 ± 20.6 years. The median age was 54.5 years (interquartile range: 31–72 years). More women participated in the study (86/134; 64%). The mean BMI of the sample was 25.3 kg/m^2^ ± 5.9 kg/m^2^ and median BMI was 24.7 kg/m^2^ (interquartile range: 21.3 kg/m^2^ – 27.9 kg/m^2^). The majority of the participants (101/134; 75.4%) were married and all but one were Christian. Of the sample, 116 participants (87%) were from the Ibo ethnic group. More than half of the participants (78/134; 58.2%) reported secondary school or higher as their highest educational level.

**TABLE 1:** Characteristics of the study participants (n = 134).

Variable	Characteristic	Frequency (percentage)
Age group (years)	20–30	33 (24.6)
	31–40	10 (7.5)
	41–50	16 (11.9)
	51–60	23 (17.2)
	61–70	16 (11.9)
	71–80	30 (22.4)
	81–90	5 (3.7)
	≥91	1 (0.8)
Marital status	SingleMarried	33 (24.6) 101 (75.4)
Gender	Male	48 (35.8)
	Female	86 (64.2)
Ethnicity	Ibo Hausa Other†	116 (86.6) 3 (2.2) 15 (11.2)
Religion	Christianity	133 (99.2)
	Islam	1 (0.8)
Educational level	None or pre-school Primary school Secondary school Tertiary education	24 (17.9) 32 (23.9) 44 (32.8) 34 (25.4)
Village	Idinisagba	45 (33.6)
	Umuodafe	67 (50.0)
	Ogboli	22 (16.4)

† **Other ethnic groupings include Urhobo, Isoko, Ozoro, Tiv, Efik and Ibibio.**

The overall prevalence of hypertension in the Ibusa community was 44% (Table 2), as determined according to the reported hypertensive status or measurements during the survey.

**TABLE 2:** Overall prevalence of hypertension in the study sample (n = 134).

Classification	Prevalence (%)
Known hypertensive patient†	34.3
Hypertension based on observed measurement‡	33.6
Hypertension based on combination of both criteria	44.0

† Agreement between criteria (a) and (b): 79.9% or kappa score of 0.55 (moderate agreement).

‡ If hypertension was confirmed, either through self-reporting (a) or measurement during the survey (b), participants were classified as hypertensive.

Table 3 shows the prevalence of hypertension organised according to demographic variables. A significantly higher prevalence was seen amongst female participants than amongst male participants (52.3% vs. 29.2%; *P* = 0.011). Prevalence of hypertension increased significantly with age (P < 0.001), and was significantly higher in the Ogboli village (81.2%; *P* < 0.001) than in Idinisagba (37.8%) or Umuodafe (35.8%). Prevalence of hypertension was significantly higher amongst married individuals than amongst single participants (56.4% vs. 6.1%; *P* < 0.001).

**TABLE 3:** Results of Pearson’s chi-square (x^2^) analysis of prevalence of hypertension organised according to demographic variables.

Variable	Characteristic	Number of hypertensive participants (prevalence %)	P-value†
Age group (years)	20–30	0 (0.0)	< 0.001
	31–40	3 (30.0)	
	41–50	9 (56.3)	
	51–60	13 (56.5)	
	61–70	11 (68.8)	
	71–80	18 (60.0)	
	81–90	5 (100.0)	
	>91	0 (0.0)	
Marital status	Single	2 (6.1)	< 0.001
	Married	57 (56.4)	
Gender	Male	45 (52.3)	0.011
	Female	14 (29.2)	
Ethnicity	Ibo	58 (50)	0.001
	Hausa	0 (0.0)	
	^b^Other	1 (6.7)	
Religion	Christianity	59 (44.4)	-J
	Islam	0 (0.0)	
Educational level	None or pre-school	15 (62.5)	0.013
	Primary school	19 (59.4)	
	Secondary school	14 (31.8)	
	Tertiary education	11 (32.4)	
Village	Idinisagba	17 (37.8)	< 0.001
	Umuodafe	24 (35.8)	
	Ogboli	18 (81.8)	

† Fisher’s exact test.

‡ Not applicable, as only one individual in Islam category.

In Table 4, bivariate and multivariate logistic regression analyses were used to identify risk factors associated with hypertension after adjustment for potential confounding from other variables. After multivariate adjustment, increasing age and BMI, as well as high salt intake, remained significantly associated with hypertension *(P* < 0.001). No significant difference in risk was found when comparing Umuodafe to Idinisagba as the reference village. However, participants from Ogboli showed markedly increased odds (risk) of hypertension compared to those from Idinisagba after multivariate adjustment *(P* < 0.001). Ibo ethnicity was associated with higher risk of hypertension after multivariate adjustment; however, this finding was not statistically significant at the 5% level *(P* = 0.062).

**TABLE 4:** Bivariate and multivariate logistic regression analyses of factors associated with hypertension.

Factor	Bivariate		Multivariate	
	
	OR (95% CI)	*P*	OR (95% CI)	*P*
**Demographic factors**				
Age	1.78 (1.43, 2.22)	< 0.001	2.03 (1.34, 3.06)	0.001
Male gender	0.38 (0.18, 0.8)	0.011	0.63 (0.19, 2.02)	0.435
Ethnicity (Ibo vs Hausa/other)	17.00 (2.19, 131.97)	0.007	19.17 (0.87, 424.17)	0.062
**Educational status**				
Primary school or less	1	-	1	-
Secondary school or higher	0.31 (0.15, 0.63)	0.001	0.94 (0.28, 3.24)	0.928
**Marital status**				
Married vs single†	20.08 (4.56, 88.48)	< 0.001	-	-
**Genetic risk factors**				
Hypertension in parents	0.84 (0.39, 1.8)	0.652	-	-
Family history of hypertension	1.04 (0.9, 1.21)	0.596	-	-
**BMI category**				
Normal	1	-	1	-
Underweight	2.10 (0.48, 9.27)	0.327	0.67 (0.07, 6.13)	0.724
Overweight	2.00 (0.89, 4.50)	0.094	4.25 (1.22, 14.83)	0.023
Obese	3.93 (1.43, 10.81)	0.008	8.17 (1.99, 33.5)	0.004
**Lifestyle risk factors**				
Abdominal circumference‡	1.06 (1.03, 1.09)	< 0.001	-	-
High salt intake	1.59 (0.79, 3.20)	0.193	3.43 (1.03, 11.47)	0.045
High alcohol intake	1.10 (0.55, 2.21)	0.782	-	-
Diet high in cholesterol or fat	1.78 (0.88, 3.63)	0.111	1.60 (0.54, 4.75)	0.396
Little physical activity	1.14 (0.55, 2.39)	0.72	-	-
Smoking	0.74 (0.37, 1.50)	0.406	-	-
Stress	1.24 (0.59, 2.59)	0.576	-	-
Noise	1.23 (0.61, 2.48)	0.554	-	-
**Village**				
Idinisagba	1	-	1	-
Umuodafe	0.92 (0.42, 2.01)	0.833	2.36 (0.74, 7.55)	0.146
Ogboli	7.41 (2.15, 25.61)	0.002	110.5 (8.74, 1397.41)	< 0.001

BMI, body mass index; CI, confidence interval; OR, odds ratio.

† Removed from the final multivariate model owing to a high variance inflation factor (> 20).

‡ Removed from the final multivariate model owing to a high correlation (co-linearity) with BMI (Pearson correlation coefficient = 0.724; *P* < 0.05).

**FIGURE 2:**
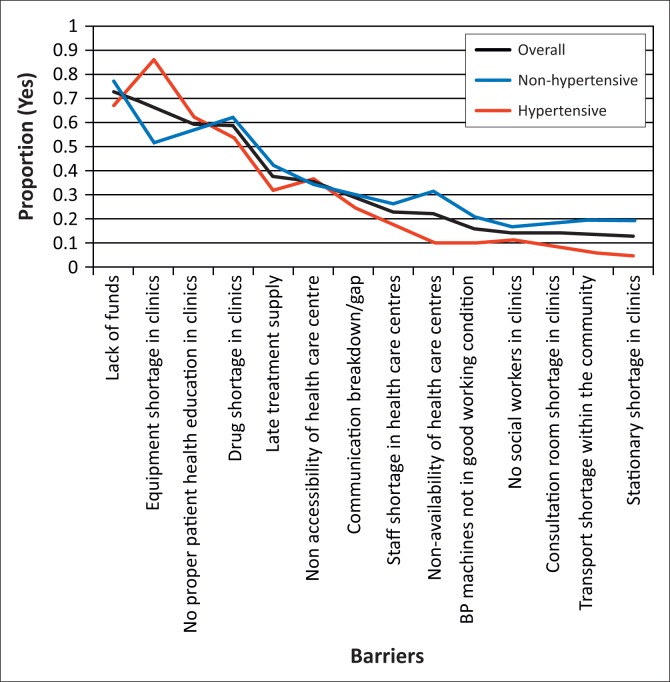
Reported barriers for management of hypertension.

Table 5 shows the reported obstacles to the management of hypertension. The obstacles most frequently reported by the study participants were lack of funds (73.1%), equipment shortage in clinics (67.2%), insufficient patient health education in clinics (59.7%) and drug shortages in clinics (59.0%). Lack of public transport within the community (14.2%) was one of the least-frequently reported obstacles. Figure 2 shows the percentage of affirmative responses with regard to possible barriers to managing hypertension in descending order. Separate curves are shown for three categories of the population (overall, non-hypertensive and hypertensive).

**TABLE 5:** Barriers to management of hypertension reported by respondents (n = 134).

Barrier	Number of responses (%)
Lack of funds	98 (73.1)
Health care centres not available	30 (22.4)
Health care centres not accessible	48 (35.8)
Transport shortage within the community	19 (14.2)
Staff shortage in health care centres	31 (23.1)
Lack of communication	38 (28.4)
No social workers in clinics	20 (14.9)
Drug shortages in clinics	79 (59.0)
Equipment shortage in clinics	90 (67.2)
Late treatment supply	51 (38.1)
Equipment not in good working condition	22 (16.4)
Limited consultation rooms	20 (14.9)
Stationary shortage in clinics	18 (13.4)
Insufficient patient health education in clinics	80 (59.7)

Table 6 shows differences in the reported obstacles to the management of hypertension amongst the hypertensive patients as determined by bivariate logistic regression analysis. Equipment shortage in clinics was significantly more likely to be reported as a problem by hypertensive than non-hypertensive patients (P < 0.001).

**TABLE 6:** Differences in reported barriers to management of hypertension according to blood pressure status, as determined by bivariate regression analysis.

Barrier	OR (95% CI)	P-value
Lack of funds	0.62 (0.29, 1.33)	0.218
Health care centres not available	0.24 (0.09, 0.64)	0.004
Health care centres not accessible	1.12 (0.55, 2.28)	0.753
Transport shortage within the community	0.29 (0.09, 0.93)	0.037
Staff shortage in health care centres	0.63 (0.27, 1.45)	0.276
Lack of communication	0.77 (0.36, 1.66)	0.504
No social workers in clinics	0.64 (0.24, 1.73)	0.380
Drug shortages in clinics	0.71 (0.35, 1.41)	0.325
Equipment shortage in clinics	5.88 (2.46, 14.08)	< 0.001
Late treatment supply	0.64 (0.31, 1.3)	0.217
Equipment not in good working condition	0.42 (0.15, 1.14)	0.090
Limited consultation rooms in clinics	0.49 (0.18, 1.37)	0.176
Stationary shortage in clinics	0.21 (0.06, 0.78)	0.019
Insufficient patient health education in clinics	1.25 (0.62, 2.52)	0.529

CI, confidence interval; OR, odds ratio.

## Discussion

The overall prevalence of hypertension found in this study (44%) aligns with other studies in rural communities in the neighbouring West African countries.^19^ However, it appears higher than the prevalence reported in rural communities in south-western Nigeria (Osun State),^5^ south-eastern Nigeria (Abia State)^6^ and the Niger Delta region^7^ (26.4%, 22.5% and 20.2%), respectively. These differences may be attributed to the sample population used in the various studies, especially with regard to age. The higher age cut-off in this study (≥ 18 years vs. ≥ 15 years in comparative studies^4,5^) may explain the higher prevalence of hypertension, as it has been shown that blood pressure increases steadily with age, irrespective of gender.^4^ There was also a markedly increased likelihood (risk) for hypertension in Ogboli village compared with other villages. This, and the overall high prevalence, may be attributed to the different cultural behaviours, for example, diets that include consumption of traditional foods such as palm kernel soup, which is a high-cholesterol food.

Beside factors that can be attributed to culture, gender, age, BMI and some lifestyle factors are well known to increase the risk for developing high blood pressure. In this study, the prevalence of hypertension was found to be higher in female participants than in male participants (29.2% vs. 52.3%), unlike findings in other studies, globally and within Nigeria, which showed a higher prevalence of hypertension in men than in women.^5^,^7^,^19^ A high prevalence of hypertension in female respondents was also reported previously in a study in Abia State in south-eastern Nigeria.^6^ In line with previous research,^7^ our study also revealed that hypertension prevalence was significantly higher in married respondents that in unmarried individuals. The issue of family, and family burden, in relation to blood pressure cannot be over-emphasised. This may be attributed to increased responsibilities or social stresses faced by married participants.^7,20^ None of the younger participants (<30 years of age) in this study population were hypertensive. This may be because hypertension in this age demographic is biologically less common.

Multivariate logistic regression indicated a significant positive association between hypertension and increasing age, as well as BMI status (overweight or obese). In this study, age, BMI and high salt intake were the strongest predictors or risk factors for hypertension. This is consistent with findings from previous studies.^5,6,7^ Increasing age, overweight and obesity are well-known risk factors for development of high blood pressure.^4,5,6,7,21,22,23,24,25,26,27^ The association between high salt intake and hypertension is well established.^28^ High salt intake in this study population is likely due to the local diet in these communities. Delta State is a riverine area that is rich in fish, and the main method of preservation is salting and smoking.

This study also revealed a number of obstacles to the management of hypertension. Amongst hypertensive patients, the most notable obstacle reported was equipment shortage in clinics (e.g. X-ray and echocardiography machines). These equipment shortages also place a constraint on health workers’ ability to do their work properly. In comparison, studies in south-eastern Nigeria^11^ and in the Limpopo province of South Africa,^9^ reported financial constraints and non-compliance with treatment, lack of public transport, staff shortage, drug shortage and late treatment supply from the hospital dispensary as obstacles to managing hypertension in local health care centres.

### Limitations

Determining the ages of participants was problematic as some could not remember their dates of birth or had lost their birth certificates during the Nigerian Civil War (Biafra). We used a guide sheet of important dates and historic events to estimate their ages. Given study design and sampling strategy, one should be cautious when generalising these findings (external validity) to other settings in Delta State. The findings are therefore only transferable to other settings with similar population, behavioural and economical characteristics. However, other rural settings within Delta State are likely to be similar to those in the current study, which should thus increase the external validity. Further studies should be conducted in other settings within Delta State and Nigeria to validate the findings of the current study.

## Conclusion

With the overall prevalence of hypertension in rural settings ranging from 13.5% to 46.4% in Nigeria, the observed hypertension prevalence (44%) is considered high in this rural community. There was also a markedly increased risk of hypertension amongst participants from the Ogboli village compared to those from the other two villages. Our findings indicate that high blood pressure and its management are an important public health problem, even in rural African settings. In view of the scarcity of resources and facilities in many developing settings, activities aimed at controlling high blood pressure, especially in rural settings, have to compete with many other pressing health needs. However, the considerable morbidity and mortality associated with high blood pressure emphasise that measures should be taken to reduce these risk factors to improve health outcomes. This may be achieved by adopting and promoting simple and cost-effective initiatives such as body weight control and increased physical activity, reducing smoking (especially amongst rural men) and reducing salt intake. Nutritional education programmes on healthy diets (low-cholesterol and low-salt diets) organised in the community may be helpful. Health policies in community settings should give particular consideration to obstacles such as those that exist at staff and administrative levels. More intervention studies are recommended to determine the factors associated with geographical heterogeneity (higherrisk areas). This will have implications for policy makers with regard to re-directing resources for the management of hypertension in high-risk populations.
